# Genome Sequence and Annotation of Bacteriophage Tokki Isolated on Arthrobacter globiformis B-2979

**DOI:** 10.1128/mra.00803-22

**Published:** 2022-09-19

**Authors:** Alicia Aguilera Marti, Nicholas D. Bullock, Samuel B. Everett, Tonya C. Bates, Ellen M. Wisner

**Affiliations:** a Department of Biological Sciences, University of North Carolina at Charlotte, Charlotte, North Carolina; Portland State University

## Abstract

*Arthrobacter* phage Tokki is a siphovirus isolated from soil in River Falls, Wisconsin. The genome contains 57,652-bp encoding 98 proteins, of which 23 were assigned a function. Tokki’s genome structure and content is typical of other AU2 subcluster phages, except for the lack of an identifiable acetyltransferase.

## ANNOUNCEMENT

Phages can be used to address increasing antibiotic resistance (1). Although Tokki is not currently a candidate, it expands the collection of annotated bacteriophages informing phage therapy efforts. Phage Tokki was isolated by students participating in the Science Education Alliance-Phage Hunters Advancing Genomics and Evolutionary Science (SEA-PHAGES) program (https://seaphages.org/) at the University of Wisconsin-River Falls using standard procedures (2). Briefly, moist soil from River Falls, Wisconsin (44.850147N, 92.624343W) was washed with peptone-yeast extract-calcium (PYCa) medium, filtered (0.22-μm pore size), and inoculated with Arthrobacter globiformis strain B-2979 with shaking at 28°C. After 2 days, the culture was filtered, diluted, and purified through two rounds of plating in top agar with A. globiformis at 28°C. After 24 h, Tokki exhibits clear plaques ~1 mm in diameter. Negative-stain transmission electron microscopy revealed Tokki to be a siphovirus with a capsid diameter of 59.6 nm and a tail length of 180.9 nm (*n* = 1).

DNA was isolated from phage lysate with the Promega Wizard DNA cleanup kit, prepared for sequencing using the NEB Ultra II kit, and sequenced using an Illumina MiSeq (MiSeq reagent kit v3), yielding ~624,995-bp 150 single-end reads that provided ~54-fold genome coverage. Untrimmed reads were assembled using Newbler version 2.9 (3) and finished with Consed version 29 (4, 5). Tokki has a 57,652-bp linear genome with 3′ single-stranded overhangs (CGCCGGCCC) and a GC content of 50.3%. Tokki is classified as a subcluster AU2 bacteriophage using previously described criteria (6, 7).

The genome was autoannotated using Glimmer 3.02 (8), GeneMark 2.5p (9), and Aragorn version 1.1 (10) embedded in DNA Master v2700 (http://cobamide2.bio.pitt.edu). Starterator v403 (http://phages.wustl.edu/starterator/), Phamerator 459 (11), and BLASTp 2.11.0 (12) were used to refine start sites. BLASTp 2.11.0 (12), HHPRED 20200717 (13), SOSUI (14), and TMHMM 2.0 (15) were used to assign gene function. While no tRNAs were identified, 98 rightward transcribed genes were identified, of which 23 were assigned a function. PECAAN 20210219 (http://discover.kbrinsgd.org) was used to review the annotation. All software programs used default settings.

Currently, Tokki is the largest phage in the AU2 subcluster (6) with the highest GC content (50.3%). Tokki’s protein-coding genes have predicted functions associated with DNA packaging and replication (genes 2, 7, 62, 79, 83, 86, 87, and 95 to 97), structure (genes 12, 14, 16, 17, 19, 22, 23, 26 to 28, 29, and 31), and lysis (gene 3). Tokki contains unique features found in other AU2 phages, including the predicted fusion of the major capsid protein, scaffold protein and capsid maturation protein genes (gene 14); two genes are predicted that encode major tail proteins (genes 17 and 19); and the predicted tape measure protein gene (gene 27) is located in between two minor tail proteins. Interestingly, Tokki does not appear to contain acetyltransferase, which is present in all other AU2 phages.

### Accession numbers.

Tokki can be accessed in GenBank with accession number OK310503.1 and Sequence Read Archive (SRA) number SRX14485097.
